# Vibration‐Mediated Recovery of Irradiated Osteocytes and Their Regulatory Role in Breast Cancer Bone Metastasis

**DOI:** 10.1002/adhm.202501689

**Published:** 2025-10-15

**Authors:** Xin Song, Kimberly Seaman, Amel Sassi, Chun‐Yu Lin, Tiankuo Chu, Liyun Wang, Yu Sun, Lidan You

**Affiliations:** ^1^ Department of Mechanical and Industrial Engineering University of Toronto Toronto ON M5S 3G8 Canada; ^2^ Institute of Biomedical Engineering University of Toronto Toronto ON M5S 3G9 Canada; ^3^ Department of Mechanical Engineering University of Delaware Newark DE 19716 USA; ^4^ Department of Mechanical and Materials Engineering Queen's University Kingston ON K7L 3N6 Canada

**Keywords:** breast cancer, mechanobiology, microfluidics, osteocytes, radiotherapy

## Abstract

Radiotherapy is a cornerstone of breast cancer treatment, but it can unintentionally damage bone, causing bone loss and pain, with no currently effective therapeutic strategy available. While chemically mediated radioprotection is extensively studied, mechanically mediated radioprotection remains underexplored. Given its safety and efficacy, this work examines the potential of low‐magnitude, high‐frequency (LMHF) vibration as a non‐invasive intervention to protect irradiated bone, focusing on osteocytes—the primary mechanosensors and regulators whose functions extend to modulating breast cancer bone metastasis. These results demonstrate that LMHF vibration (0.3 g, 60 Hz, 1 h) mitigates osteocyte apoptosis and upregulates cytoskeletal markers following 8 Gy irradiation. LMHF vibration applied 1 h per day over 3 days restores the regulatory function of irradiated osteocytes in controlling breast cancer extravasation in a microfluidic platform. A combined approach integrating vibration with radiotherapy further reduces cancer invasion and extravasation, demonstrating a compound effect. RNA sequencing (RNA‐seq) analysis reveals that this osteocyte‐mediated regulation is possibly driven by the Wnt signaling pathway. These findings highlight the potential of LMHF vibration in enhancing radiotherapy efficacy by protecting osteocytes and reducing breast cancer metastasis, underscoring the promise of a non‐invasive mechanical intervention in preserving bone health and optimizing cancer treatment outcomes.

## Graphical abstract

Radiotherapy damages bone and disrupts osteocyte function, yet mechanically mediated protection remains largely unexplored. This study demonstrates that low‐magnitude, high‐frequency (LMHF) vibration mitigates irradiated osteocyte apoptosis, restores their ability to regulate breast cancer extravasation, and acts synergistically with radiotherapy to reduce metastasis, suggesting a novel mechanical approach to protect bone and enhance cancer treatment outcomes.

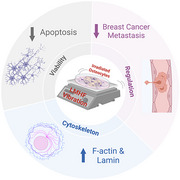

## 1 Introduction

Breast cancer remains a leading cause of mortality worldwide. It arises from mutations in breast cells, leading to uncontrolled growth and the spread of cancer to other organs. Bone is a common secondary site for breast cancer metastasis, with ≈75% of advanced‐stage breast cancer patients suffering from bone metastases.^[^ ^1^ ^]^ Metastasized cancer cells disrupt bone remodeling, leading to incurable bone lesions. As such, radiotherapy is a standard intervention for both localized and metastatic breast cancer, functioning by killing cancer cells and reducing tumor size.^[^ ^2^, ^3^ ^]^ Nearly 70% of breast cancer patients receive radiotherapy at some point during their treatment.^[^ ^4^, ^5^ ^]^ The effectiveness of radiotherapy—both as a standalone treatment and in combination with other therapies such as surgery and chemotherapy—has markedly improved patient quality of life and reduced mortality rates.

The effects of radiotherapy can extend beyond malignant cells to healthy tissues, with bone being particularly vulnerable due to its high calcium content, which absorbs 30–40% more radiation than surrounding soft tissues.^[^ ^6^ ^]^ Irradiation‐induced bone damage results in reduced bone volume, increased bone marrow adiposity, accelerated cellular apoptosis, and elevated fracture risks.^[^ ^7^, ^8^, ^9^ ^]^ This bone degradation releases embedded growth factors (e.g., transforming growth factor‐beta (TGF‐β) and insulin‐like growth factors (IGFs)), which in turn, further promote cancer colonization.^[^ ^10^, ^11^ ^]^ Additionally, irradiation increases endothelial permeability, leading to poorly vascularized bone tissue. This not only prolongs healing but also facilitates the transendothelial migration of cancer cells.^[^ ^6^, ^12^ ^]^ Consequently, the detrimental effects of irradiation (particularly at high doses) on bone can be compounded in breast cancer patients with bone metastases. To date, amifostine is the only FDA‐approved radioprotective agent, but its low effectiveness and side effects have constrained its clinical application.^[^ ^13^ ^]^ Alternative treatments can be either surgical and/or conservative using anti‐resorptive drugs such as bisphosphonates and denosumab. However, long‐term, high‐dose use of these drugs increases bone brittleness and can induce rare but severe conditions, such as osteonecrosis of the jaw.^[^ ^14^, ^15^ ^]^ Hence, there is a pressing need for improved therapeutic strategies to mitigate irradiation‐induced bone damage.

Physical exercise has been shown to benefit breast cancer patients, including reduced cancer progression and improved bone health.^[^ ^16^ ^]^ However, for patients who are bedridden, disabled, or elderly, especially those with bone metastasis or undergoing radiotherapy, engaging in intense physical exercise can be physically challenging. As a low‐intensity alternative, low‐magnitude (< 1 g), high‐frequency (> 30 Hz) (LMHF) vibration has gained increasing attention in recent years.^[^ ^17^, ^18^, ^19^, ^20^, ^21^, ^22^, ^23^, ^24^ ^]^ Evidence suggests that LMHF vibration enhances both bone quality and quantity in cancer patients^[^ ^18^ ^]^ and cancer‐bearing mice.^[^ ^19^, ^20^, ^21^ ^]^ At the cellular level, vibration has been shown to reduce bone resorption and suppress the metastatic potential of breast cancer cells.^[^ ^22^, ^23^, ^24^ ^]^ Despite these findings, the potential of mechanically mediated radioprotection remains unexplored. With its demonstrated efficacy, LMHF vibration presents a promising approach for mitigating irradiation‐induced bone damage.

An understanding of cellular responses to LMHF vibration is crucial for uncovering its radioprotective potential. Osteocytes are the most abundant cell type in bone, constituting over 90% of the bone cell population. As the primary mechanosensing cells and regulators, they detect mechanical stimuli and transduce them into biochemical signals that modulate the activities of osteoclasts and osteoblasts.^[^ ^25^, ^26^ ^]^ Beyond their fundamental role in bone maintenance, recent research has highlighted the involvement of osteocytes in bone metastasis.^[^ ^22^, ^23^, ^27^, ^28^ ^]^ Our previous study has demonstrated that LMHF vibration activates osteocytes, thereby reducing breast cancer extravasation.^[^ ^22^ ^]^ However, radiotherapy impairs osteocyte function by increasing apoptosis and altering morphology.^[^ ^29^, ^30^ ^]^ Yet there is currently a lack of research exploring potential solutions to counteract irradiation‐induced osteocyte damage.

Building on the previous findings, this study explores the potential of LMHF vibration (Figure S1, Supporting Information) in alleviating irradiation‐induced osteocyte damage and evaluates vibration's synergistic effects on breast cancer bone metastasis in combination with radiotherapy. Specifically, we examined osteocyte viability, cytoskeletal integrity, and their regulatory role in bone metastasis. We hypothesize that LMHF vibration mitigates the irradiation‐induced decline in osteocyte viability, upregulates cytoskeletal markers, and restores their regulatory role in bone metastasis following irradiation.

## 2 Results

### 2.1 Vibration Mitigated Apoptosis in Irradiated Osteocytes

**Figure 1:**
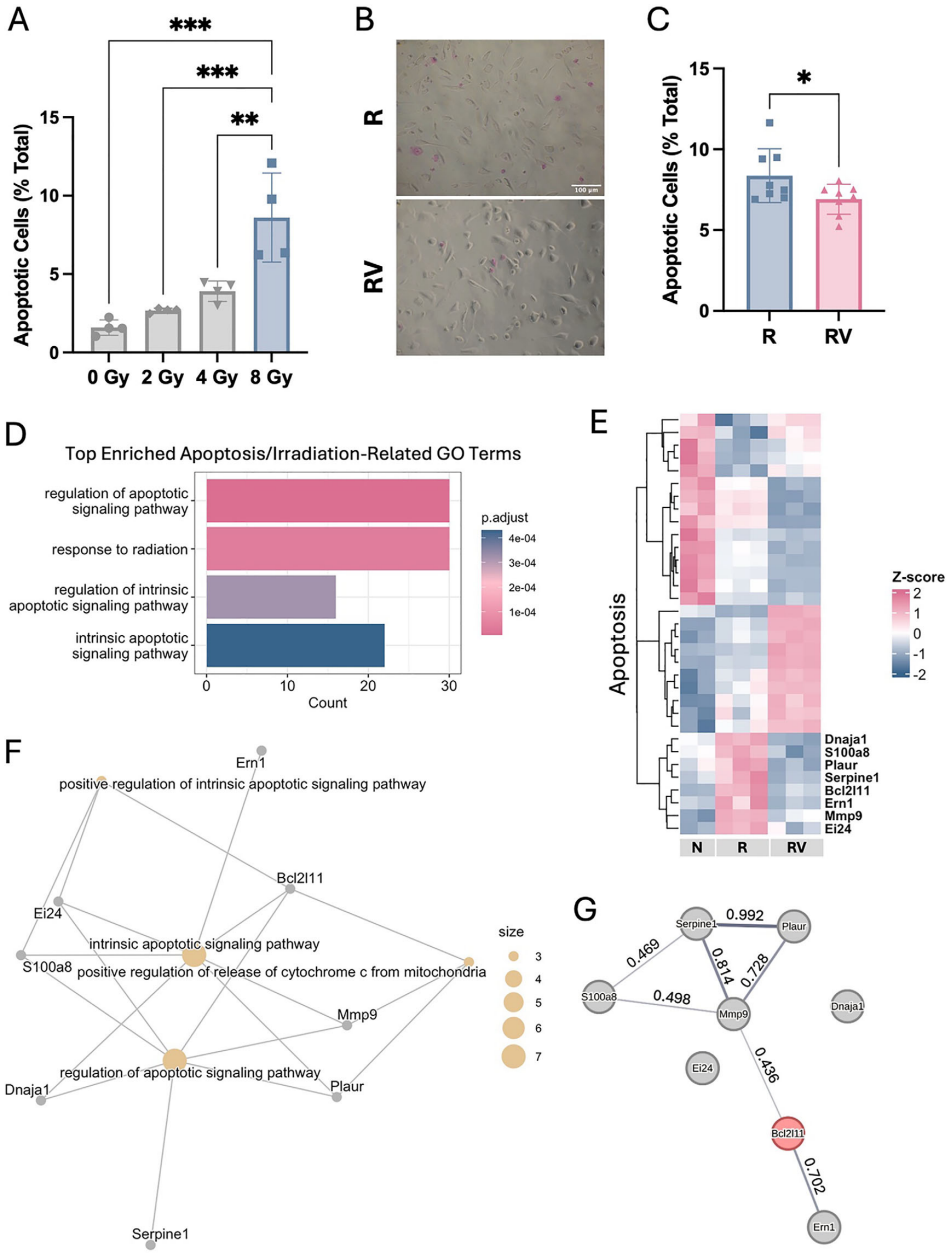
Osteocyte apoptosis. A) Number of apoptotic osteocytes normalized to the total number of osteocytes irradiated at different doses. Data presented as mean ± SD, n = 4. Significance was calculated using one‐way ANOVA with Tukey's correction (*p* < 0.05). B) Representative images of APOPercentage‐stained osteocytes showing apoptotic cells in pink. Both images share the same scale (scale bar = 100 µm). C) Number of apoptotic osteocytes normalized to the total number of osteocytes treated with vibration or static conditions following irradiation. Data presented as mean ± SD, n = 8. Significance was calculated using Student's *t*‐test (*p* < 0.05). D) Enrichment of the top apoptosis/irradiation‐related gene ontology (GO) terms. E) Heatmap of differentially expressed genes (DEGs) related to apoptosis shown in panel D. Labeled genes: irradiation‐induced expression changes that were reversed by vibration. F) Gene concept network enriched in the labeled genes. G) STRING analysis of protein–protein interactions (PPIs) among proteins encoded by the labeled genes with interaction scores. N: non‐irradiated osteocytes, R: irradiated osteocytes, RV: irradiated osteocytes treated with vibration.

Using Apopercentage stain, we compared the effects of 2, 4, and 8 Gy irradiation on osteocytes and found that apoptosis was significantly increased 2 days post‐irradiation at 8 Gy (**Figure** **1**A). This increase in apoptosis was reduced by a single 1‐h LMHF vibration at 0.3 g and 60 Hz (Figure 1B,C). Additionally, irradiation induced DNA double‐strand breaks, as indicated by γH2AX‐labeled foci (Figure S2, Supporting Information). However, vibration did not significantly alter irradiation‐induced DNA damage (Figure S2B, Supporting Information).

To assess the effects of irradiation and vibration on osteocyte gene expression, we performed RNA sequencing (RNA‐seq) and conducted a multiple‐group comparison using the irradiated group (R) as the reference, since it represents the common condition linking both contrasts (N versus R and RV versus R) (Figure S3A–C, Supporting Information). A total of 559 genes were differentially expressed (Figure S3B,C, Supporting Information). The top five enrichment terms related to apoptosis and irradiation are shown in Figure 1D, with the corresponding genes visualized in heatmaps (Figure 1E and Figure S3D, Supporting Information). To explain the apoptosis reversal observed in Figure 1C, we focused on genes whose irradiation‐induced changes were reversed by vibration and those can be functionally categorized into gene ontology (GO) terms. We found that vibration partially reversed irradiation‐induced upregulation of several apoptosis‐related genes (labeled in Figure 1E). This reversal occurred primarily through the intrinsic apoptotic signaling pathway (Figure 1F), which was activated when Bcl‐2 family pro‐apoptotic proteins permeabilized the mitochondrial outer membrane, leading to the release of cytochrome c into the cytosol.^[^ ^31^ ^]^ Our GO analysis identified *Mmp9*, *Plaur*, and *Bcl2l11* as positive regulators of cytochrome c release, and *Ei24*, *S100a8*, and *Bcl2l11* as positive regulators of the intrinsic apoptotic signaling pathway, with *Bcl2l11* serving as the common regulator (Figure 1F). These positive regulators were upregulated by irradiation, indicating activation of intrinsic apoptosis, but their expression was suppressed by vibration, suggesting a partial reversal of this apoptotic signaling (Figure 1E,F). STRING analysis further revealed that *Bcl2l11* (*BIM*) serves as a central hub connecting the proteins encoded by these reversed genes (Figure 1G), highlighting its key role in mediating the intrinsic apoptotic response. In addition, gene set enrichment analysis (GSEA) revealed that vibration downregulated the inflammatory response in irradiated osteocytes (Figure S3E, Supporting Information).^[^ ^31^ ^]^

### 2.2 Vibration Upregulated F‐Actin in Irradiated Osteocytes

**Figure 2:**
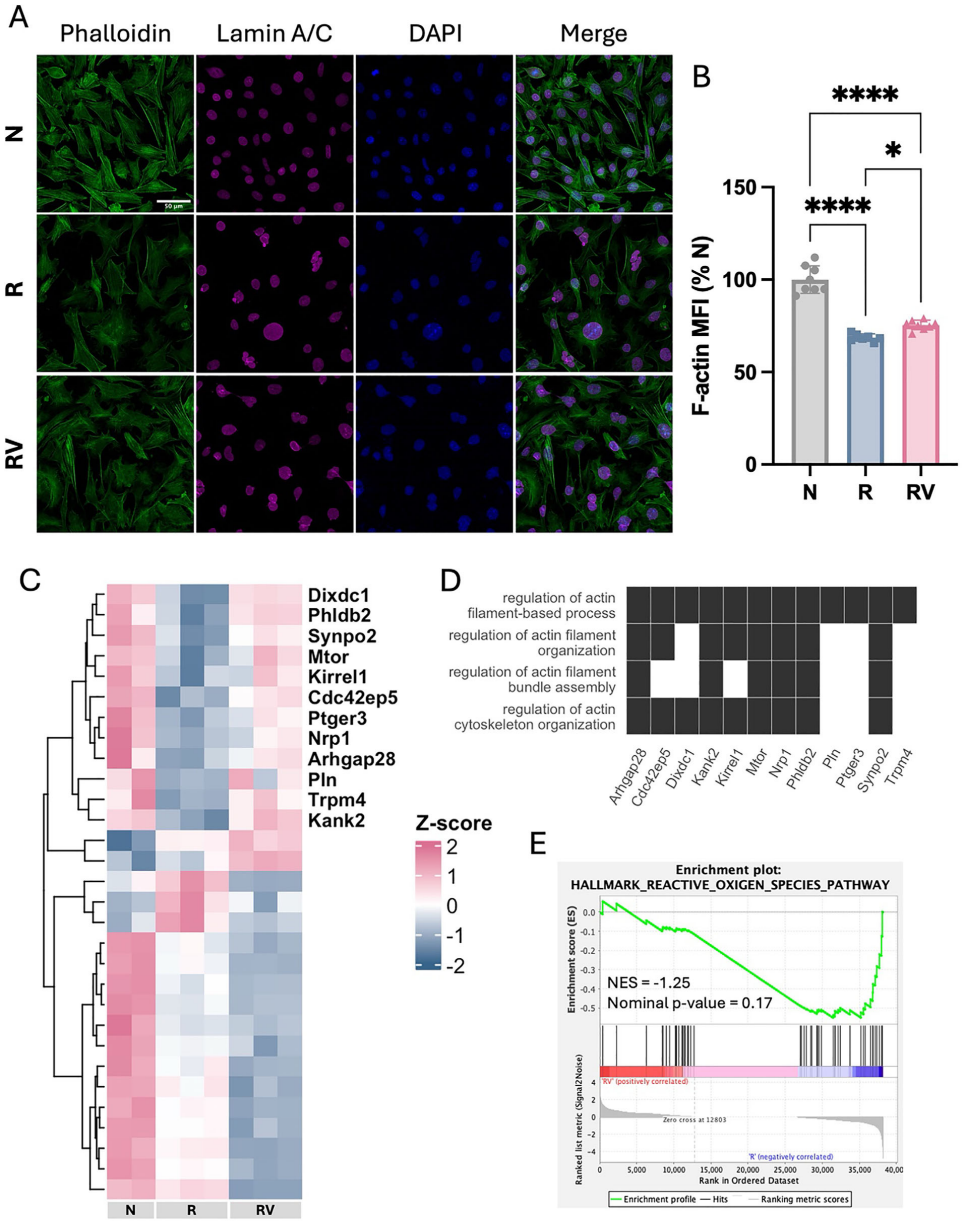
F‐actin in irradiated osteocytes. A) Immunofluorescence staining of phalloidin, lamin A/C, and DAPI. All images share the same scale (scale bar = 50 µm). B) Mean fluorescent intensity of phalloidin‐stained F‐actin. Data presented as mean ± SD, n = 8. Significance was calculated using one‐way ANOVA with Tukey's correction (*p* < 0.05). C) Heatmap of differentially expressed genes (DEGs) related to actin filaments. Labeled genes: irradiation‐induced expression changes that were reversed by vibration. D) Gene ontology (GO) classification for labeled genes. E) Gene set enrichment analysis (GSEA) of the reactive oxygen species pathway in RV relative to R. NES: normalized enrichment score. N: non‐irradiated osteocytes, R: irradiated osteocytes, RV: irradiated osteocytes treated with vibration.

The actin cytoskeleton is a dynamic network of proteins that plays a crucial role in osteocyte functions, such as cell motility, structural maintenance, apoptosis, organelle transport, and mechanosensing.^[^ ^25^, ^32^ ^]^ By staining with phalloidin after 8 Gy irradiation followed by a single bout of vibration, we found that irradiation disrupted the actin cytoskeleton by decreasing filamentous actin (F‐actin) expression, as quantified by mean fluorescence intensity (**Figure** **2**A,B), reducing cellular branching while expanding the F‐actin‐labeled cell area (Figure S4A,B, Supporting Information). In contrast, vibration slightly increased F‐actin expression compared to static conditions in irradiated osteocytes (Figure 2B). Furthermore, RNA‐seq analysis revealed that irradiation exerted an inhibitory effect by downregulating most actin‐filament‐associated genes (Figure 2C). Notably, a subset of these genes showed partial reversal upon vibration (labeled in Figure 2C). GO analysis showed that the reversed gene expression changes were involved in actin filament organization and bundle assembly (Figure 2D). In addition, our GSEA demonstrated a trend toward downregulation of the reactive oxygen species (ROS) pathway following vibration treatment (Figure 2E). This pathway is linked to oxidative stress, which has been identified as a major contributor to irradiation‐induced cytoskeletal damage.^[^ ^33^ ^]^

### 2.3 Vibration Upregulated Nuclear Lamins in Irradiated Osteocytes

**Figure 3:**
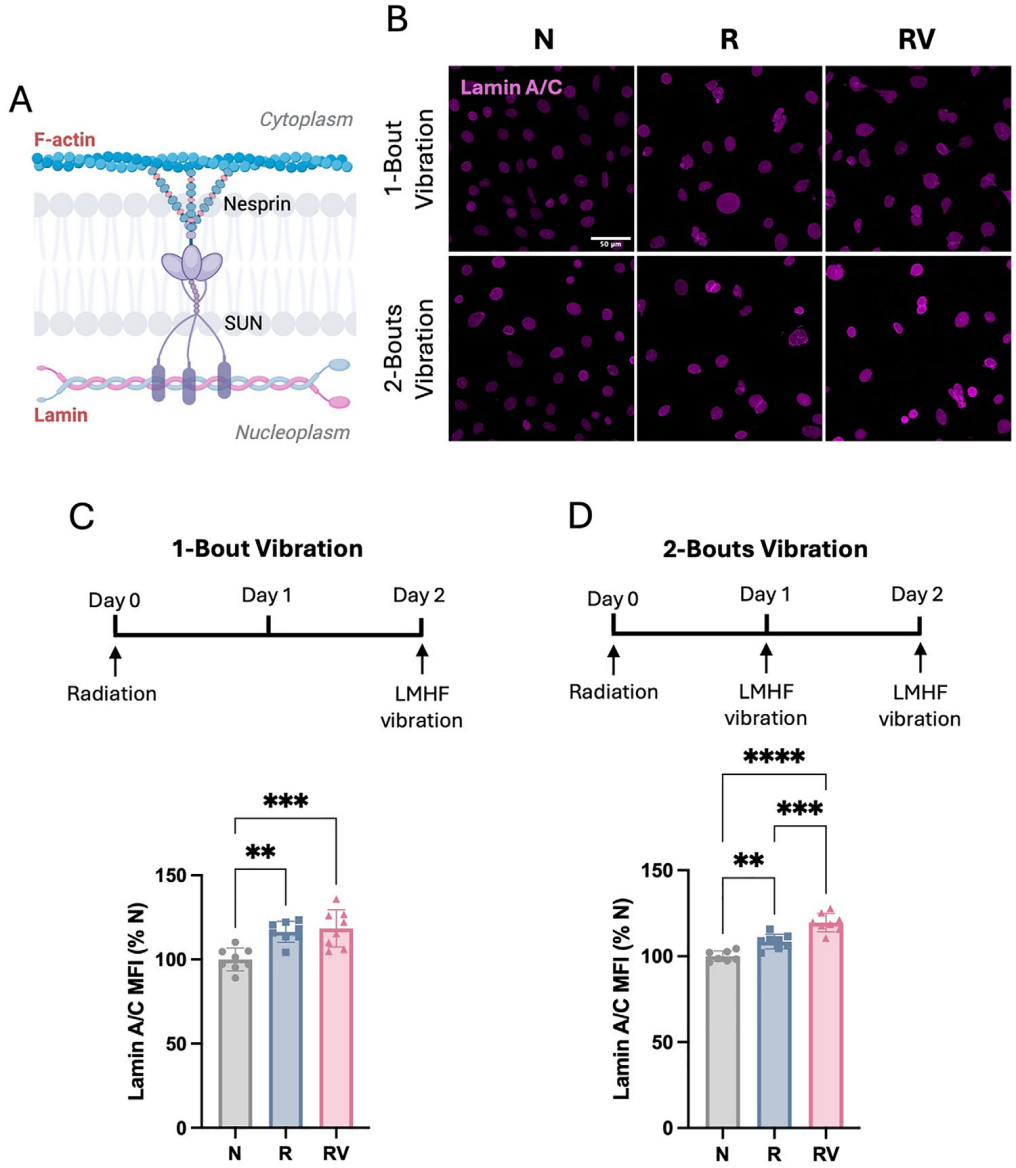
Nuclear envelope in irradiated osteocytes. A) Schematic illustration of the linker of nucleoskeleton and cytoskeleton (LINC) complex. B) Immunofluorescence staining of lamin A/C. All images share the same scale (scale bar = 50 µm). C) Timeline of one‐bout vibration and mean fluorescent intensity of lamin A/C–stained nuclear envelope. D) Timeline of two‐bout vibration and mean fluorescent intensity of lamin A/C–stained nuclear envelope. Data presented as mean ± SD, n = 8. Significance was calculated using one‐way ANOVA with Tukey's correction (*p* < 0.05). N: non‐irradiated osteocytes, R: irradiated osteocytes, RV: irradiated osteocytes treated with vibration.

F‐actin interacts with linker of nucleoskeleton and cytoskeleton (LINC) complex, which connects to lamins lining the inner nuclear envelope (**Figure** **3**A). Lamin A/C proteins are essential for maintaining nuclear integrity, facilitating DNA repair processes, and providing structural support to the nucleus. They also function as mechanosensing sites in osteocytes.^[^ ^34^, ^35^ ^]^ Our immunofluorescent staining showed increased lamin A/C expression in non‐irradiated osteocytes after two bouts of vibration (Figure S5, Supporting Information). Following 8 Gy irradiation, osteocytes displayed enlarged nuclear area with irregular morphology, as reflected by reduced roundness measured by form factor (Figure S4C, Supporting Information), consistent with reports in other irradiated cells.^[^ ^36^ ^]^ Furthermore, irradiation elevated lamin A/C expression, as quantified by mean fluorescence intensity (Figure 3B–D), and this increase was further amplified by the two‐bout vibration regimen in irradiated osteocytes (Figure 3D).

### 2.4 Vibration Recovered Irradiated Osteocyte Regulation of Breast Cancer Metastasis

Osteocytes, the primary regulators of bone homeostasis, regulate the activity of osteoclasts and osteoblasts. By culturing RAW cells in osteocyte‐conditioned media, we found that irradiated osteocytes promoted the differentiation of non‐irradiated RAW cells into osteoclasts, as shown by tartrate‐resistant acid phosphatase (TRAP) staining (Figure S6A–C, Supporting Information). When vibration was applied to irradiated osteocytes, they did not significantly alter the total number of osteoclasts (Figure S6B, Supporting Information) but did reduce the formation of multinucleated giant osteoclasts (with >10 nuclei) (Figure S6C, Supporting Information). Notably, our RNA‐seq data suggested that vibration partially reversed the irradiation‐induced downregulation of genes involved in bone development and bone growth in osteocytes (Figure S6D,E, Supporting Information).

**Figure 4:**
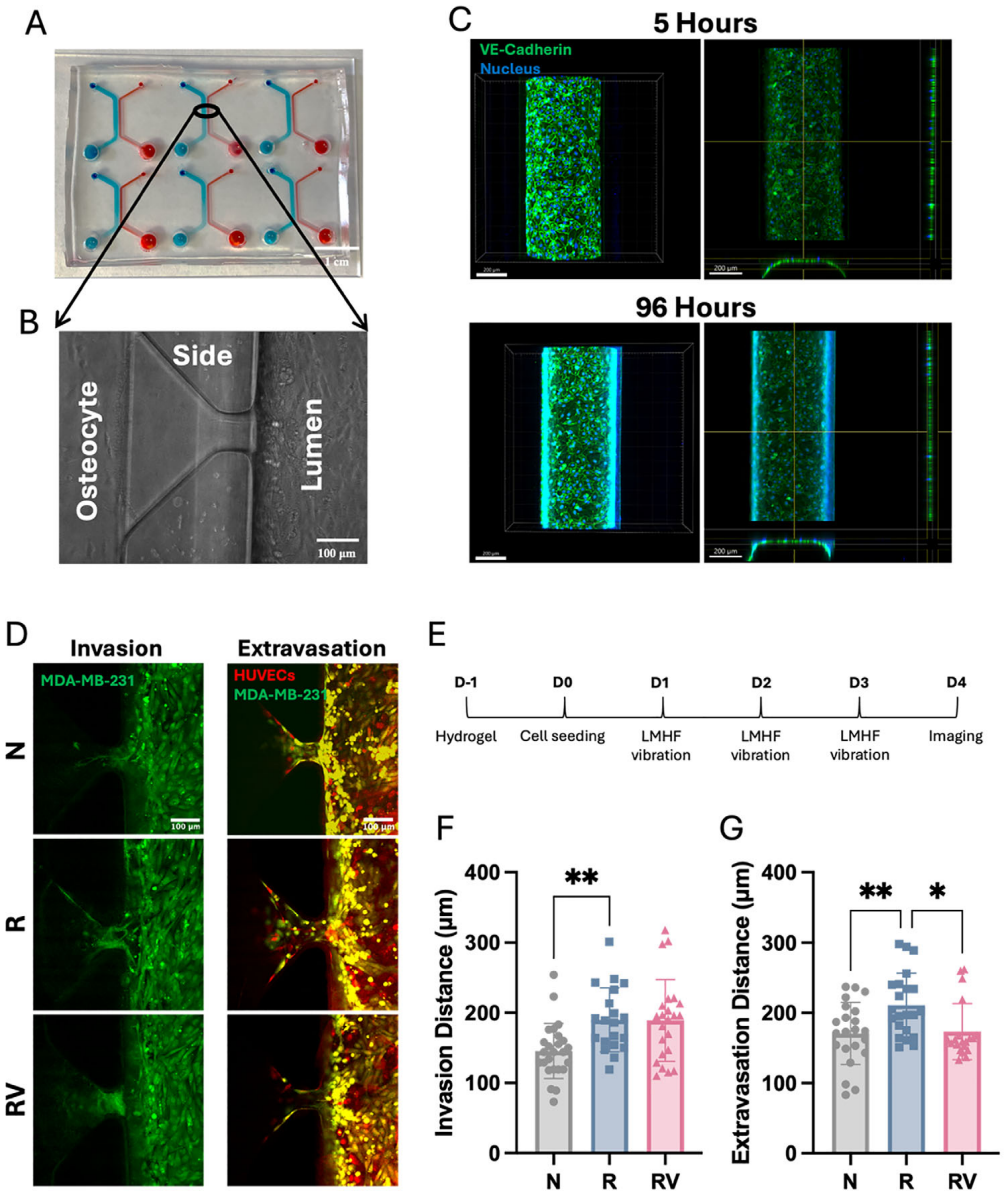
Irradiated osteocyte regulation of cancer invasion and extravasation. A) Microfluidic platform containing six microfluidic devices, each with five side channels. Osteocyte channels are shown in blue, and lumen channels in red. Scale bar = 1 cm. B) Top view of the osteocyte channel, side channel, and lumen channel. Scale bar = 100 µm. C) Immunofluorescence staining of VE‐cadherin and DAPI in HUVECs after 5 and 96 h of seeding in the device. Scale bar = 200 µm. D) Representative fluorescence images showing breast cancer (Green, CellTracker^TM^ Green) invasion and extravasation through the endothelial barrier (Red, CellTracker^TM^ Orange) co‐cultured with non‐irradiated or irradiated osteocytes in the device under static or vibration conditions. All images share the same scale (scale bar = 100 µm). E) Timeline of cancer invasion and extravasation experiments. F) Quantifications of cancer invasion distance and G) extravasation distance co‐cultured with non‐irradiated or irradiated osteocytes in the device under static or vibration conditions on Day 4. Data presented as mean ± SD, n = 25. Significance was calculated using one‐way ANOVA with Tukey's correction (*p* < 0.05). N: no irradiation, R: irradiation (on osteocytes only), RV: irradiation (on osteocytes only) and vibration.

Beyond bone maintenance, osteocytes are also involved in regulating bone metastasis. To investigate this, we utilized a microfluidic device with 3D hydrogel to mimic the bone–cancer microenvironment and study breast cancer invasion (without HUVECs) and extravasation (through a HUVEC barrier) (**Figure** **4**A,B). For validation, HUVECs formed VE‐cadherin intercellular connections within 5 h of seeding, which remained intact for up to 96 h (Day 4) (Figure 4C). In our previous research, we observed reduced breast cancer invasion and extravasation under vibration treatment only in the presence of osteocytes.^[^ ^22^ ^]^ However, irradiated osteocytes showed a diminished ability to inhibit cancer metastasis, leading to increased invasion and extravasation of non‐irradiated breast cancer cells (Figure 4D–G). Notably, applying vibration for 1 h daily over 3 days (Figure 4E) restored osteocyte regulatory function following irradiation, reducing cancer extravasation by 18% (Figure 4G).

### 2.5 Vibration Further Reduced Breast Cancer Metastasis Following Irradiation

**Figure 5:**
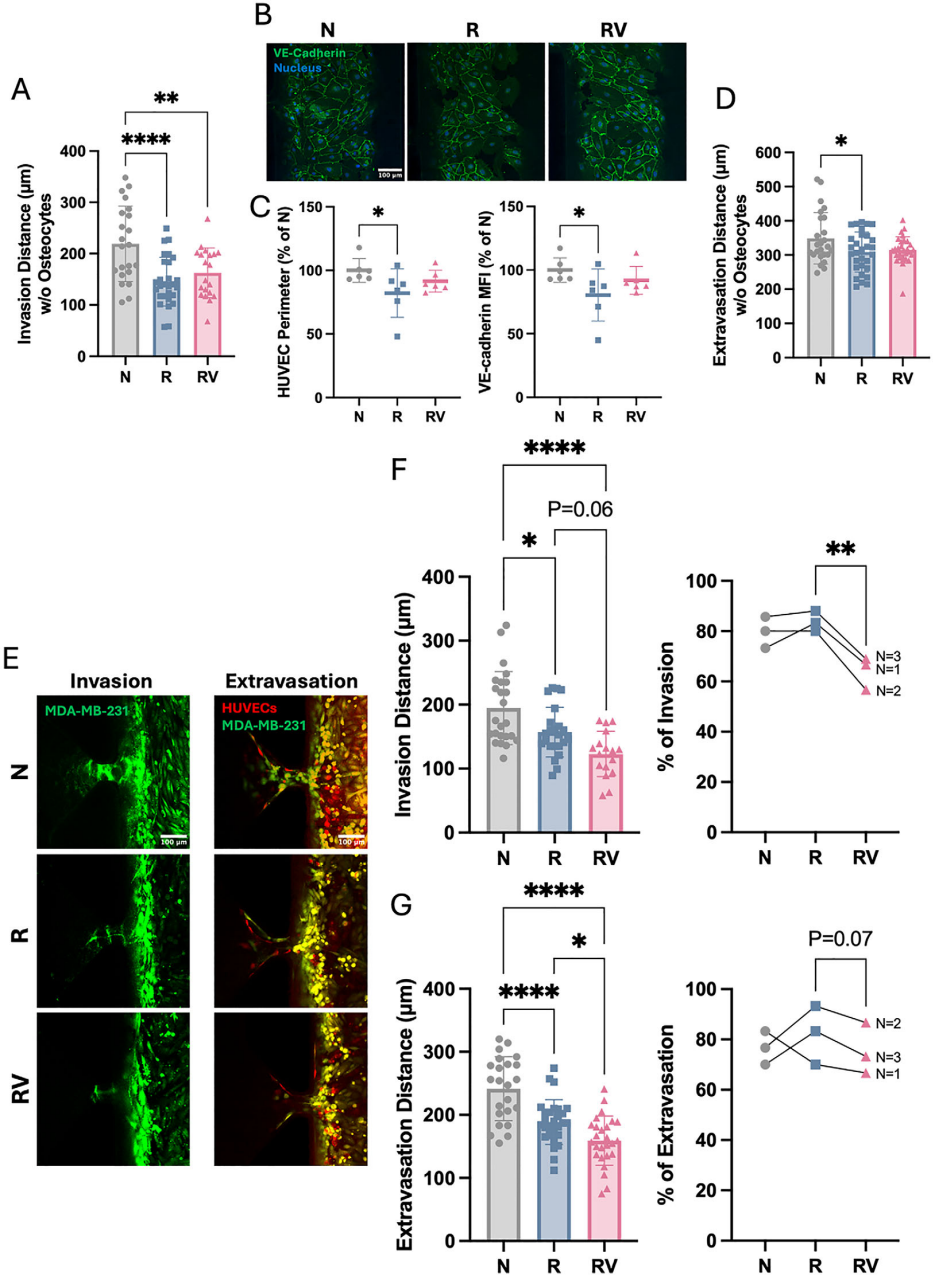
Cancer invasion and extravasation under vibration treatment following irradiation. A) Non‐irradiated or irradiated cancer invasion distance without osteocyte regulation under static or vibration conditions on Day 4, n = 25. B) Immunofluorescence staining of VE‐cadherin and DAPI in non‐irradiated or irradiated HUVECs under static or vibration conditions. All images share the same scale (scale bar = 100 µm). C) Analysis of cell perimeter and mean fluorescent intensity of VE‐cadherin in HUVECs on Day 4, n = 4. D) Non‐irradiated or irradiated cancer extravasation distance without osteocyte regulation under static or vibration conditions on Day 4, n = 25. E) Representative fluorescence images showing breast cancer (Green, CellTracker^TM^ Green) invasion and extravasation through the endothelial barrier (Red, CellTrackerTM Orange) co‐cultured with osteocytes in the device under static or vibration conditions with or without irradiation. All images share the same scale (scale bar = 100 µm). F) Quantifications of cancer invasion distance (n = 25), percentage of invasion (N = 3), G) extravasation distance (n = 25), and percentage of extravasation (N = 3) under static or vibration conditions with or without irradiation on Day 4. Data presented as mean ± SD. Invasion/extravasation distance and HUVEC analysis: significance was calculated using one‐way ANOVA with Tukey's correction (*p* < 0.05). Invasion/extravasation percentage: significance was calculated using paired Student's *t‐*test (*p* < 0.05). N: no irradiation, R: irradiation, RV: irradiation and vibration.

We further investigated the effects of irradiation on all cells in the microfluidic device, noting that localized cells are exposed to radiation in vivo. In the absence of osteocytes, irradiation decreased breast cancer cell invasion, while vibration had no direct effect on cancer invasion (**Figure** **5**A). Additionally, irradiation disrupted HUVEC intercellular junctions by reducing VE‐cadherin levels at cell–cell contact sites, which correlated with a decrease in HUVEC perimeter (Figure 5B,C). The mean fluorescence intensity of VE‐cadherin was also reduced by irradiation (Figure 5B,C), suggesting compromised barrier integrity. When breast cancer cells were seeded into an HUVEC barrier, irradiation (on both cancer cells and HUVECs) resulted in an overall reduction in cancer extravasation, while vibration had no significant effect (Figure 5D). Thus, vibration did not directly affect breast cancer invasion or extravasation in the absence of osteocytes.

In co‐culture with osteocytes, irradiation reduced both cancer invasion and extravasation distances (Figure 5E–G). Subsequent vibration treatments (Figure 4E) tended to further reduce invasion distance and significantly decreased extravasation distance (Figure 5F,G). Due to irradiation of cancer cells, some side channels remained uninvaded. Quantification of invasion and extravasation percentages revealed that vibration significantly reduced the percentage of invasion and also showed a trend toward reducing the percentage of extravasation following irradiation (Figure 5F,G).

### 2.6 Mechanism Underlying Osteocyte Regulation of Cancer

**Figure 6:**
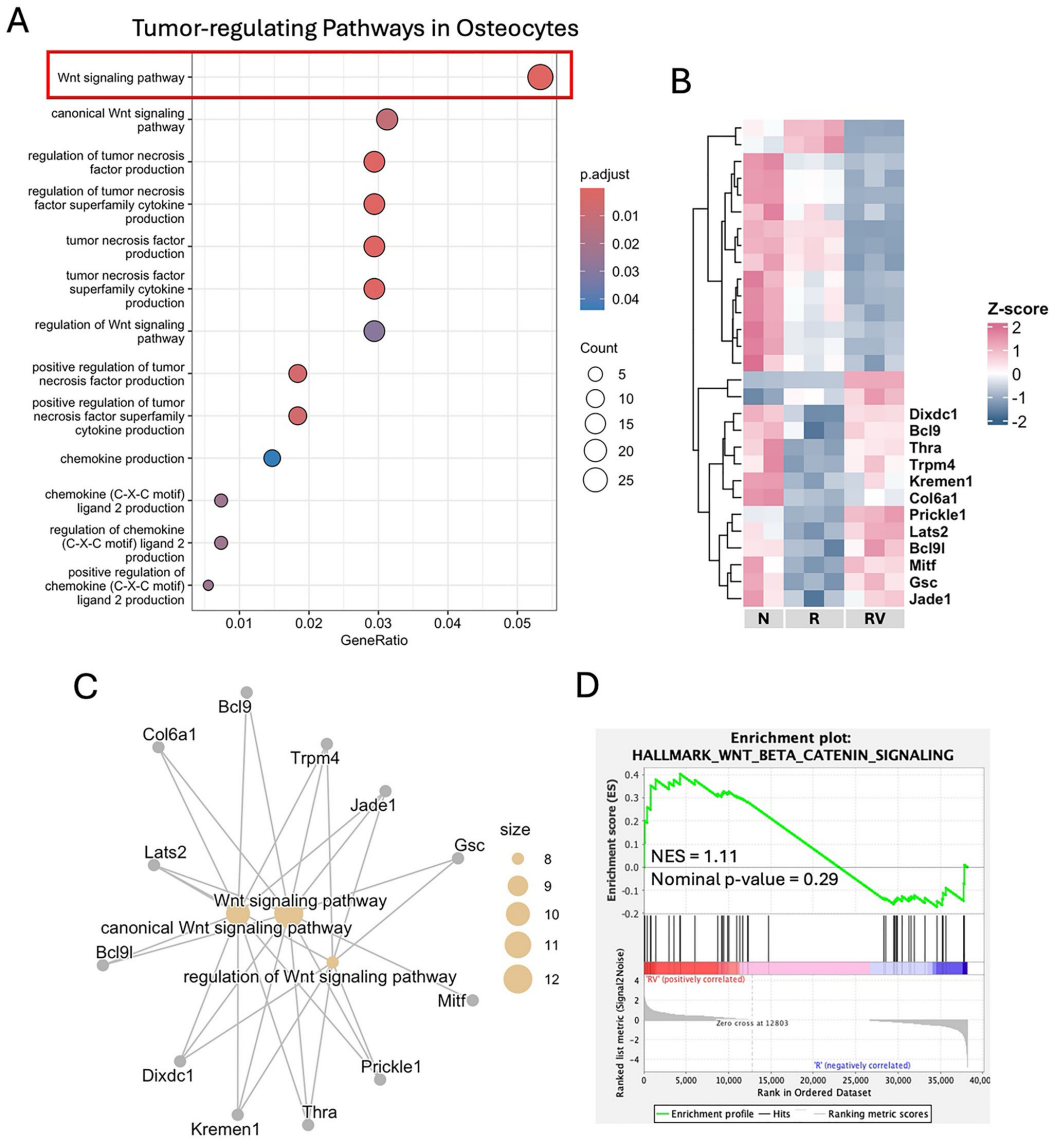
RNA‐seq analysis depicting osteocyte regulation of cancer. A) Enriched gene ontology (GO) terms related to osteocyte‐mediated regulation of cancer behaviors. A red box highlights the Wnt signaling pathway. B) Heatmap plot of differentially expressed genes (DEGs) related to the Wnt signaling pathway. Labeled genes: irradiation‐induced expression changes that were reversed by vibration. C) Gene concept network enriched in labeled genes from panel B. D) Gene set enrichment analysis (GSEA) of the Wnt and β‐catenin signaling in RV relative R. NES: normalized enrichment score. N: non‐irradiated osteocytes, R: irradiated osteocytes, RV: irradiated osteocytes treated with vibration.

Among the various tumor‐regulating pathways in osteocytes, such as tumor necrosis factors, chemokines, and cytokines, the Wnt signaling pathway was most prominently enriched (highlighted by the box in **Figure** **6**A), indicating that a greater proportion of Wnt pathway‐associated genes were differentially expressed in response to irradiation and vibration compared with other pathways. Closer examination showed that irradiation downregulated Wnt‐related genes, while approximately half of them could be upregulated by vibration (labeled in Figure 6B), thereby partially reversing the irradiation‐induced suppression. The gene concept network further illustrated the connectivity between the reversed Wnt‐related genes and GO terms (Figure 6C). To assess the overall impact of vibration on the Wnt signaling pathway, we performed GSEA, which revealed a positive enrichment trend following vibration treatment (Figure 6D), suggesting partial reactivation of Wnt signaling in response to vibration.

**Figure 7:**
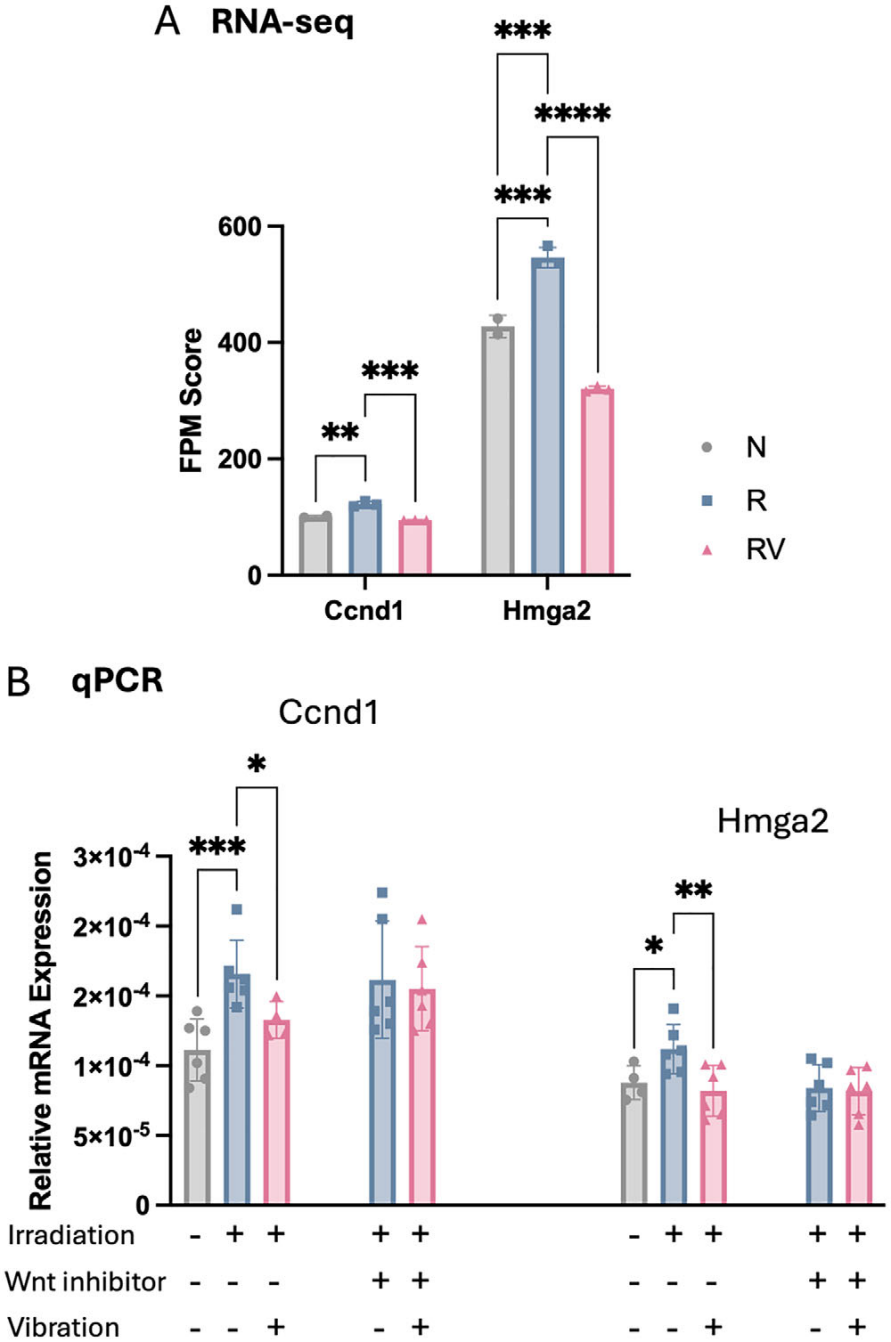
Tumor‐promoting genes in irradiated osteocytes. A) Fragments per million mapped reads (FPM) of the tumor‐promoting genes Ccnd1 and Hmga2. N: non‐irradiated osteocytes, R: irradiated osteocytes, RV: irradiated osteocytes treated with vibration. B) mRNA expression of Ccnd1 and Hmga2 in non‐irradiated or irradiated osteocytes under static or vibration conditions, confirming the RNA‐seq data. Data presented as mean ± SD, n = 6. Significance was calculated using one‐way ANOVA with Tukey's correction (*p* < 0.05). Relative mRNA expression of Ccnd1 and Hmga2 in irradiated osteocytes treated with a Wnt inhibitor under static or vibration conditions. 18S was used as the housekeeping gene.

In addition to its effect on tumor‐regulating pathways, vibration directly influenced the expression of tumor‐related genes in irradiated osteocytes. We focused on breast cancer–related genes whose irradiation‐induced changes could be reversed by vibration. RNA‐seq analysis revealed that vibration counteracted irradiation‐induced upregulation of the tumor‐promoting genes *Ccnd1* and *Hmga2* (**Figure** **7**A), a finding further confirmed by quantitative polymerase chain reaction (qPCR) (Figure 7B). Specifically, irradiation upregulated *Ccnd1* and *Hmga2*, whereas vibration significantly reduced their expression (Figure 7). To investigate the role of Wnt signaling in the expression of tumor‐promoting genes, we inhibited the pathway in irradiated osteocytes using CCT036477.^[^ ^37^, ^38^ ^]^ Wnt inhibition impaired the osteocytic response to vibration, attenuating the vibration‐induced downregulation of *Ccnd1* and *Hmga2* (Figure 7B).

## 3 Discussion

Radiotherapy is essential in breast cancer treatment, but its efficacy is limited by unintended damage to bone, including osteocytes, which are crucial for maintaining bone health and regulating bone metastasis. In this study, we investigated the potential of LMHF vibration to mitigate irradiation‐induced osteocyte damage and explored its therapeutic synergy with radiotherapy in bone metastasis. Our results showed that LMHF vibration reduced osteocyte apoptosis and alleviated the loss of F‐actin expression following irradiation. Using a microfluidic platform, we demonstrated that vibration restored osteocyte‐mediated regulation of breast cancer extravasation, potentially through the Wnt signaling pathway. Notably, the combination of vibration and irradiation cumulatively suppressed breast cancer invasion and extravasation, highlighting the therapeutic potential of LMHF vibration as an adjunct to radiotherapy in slowing breast cancer progression.

Irradiation dosage should be individualized for patients and tailored to the therapeutic purpose. For whole breast radiotherapy, patients typically receive a hypofractionated dose ranging from 40 to 50.4 Gy, delivered in 15 to 28 fractions, with each fraction ranging from 1.8 to 2.67 Gy.^[^ ^2^ ^]^ As the animal model size decreases, the dosage is correspondingly reduced. In rat models, a total dose of 24 Gy, administered at 8 Gy/day over 3 days, is considered equivalent to a human dose of 48 Gy.^[^ ^9^, ^39^ ^]^ When transitioning to cellular models, researchers commonly use single doses ranging from 2 to 10 Gy to study the effects of irradiation on breast cancer cells,^[^ ^12^, ^40^, ^41^ ^]^ endothelial cells,^[^ ^12^ ^]^ and osteocytes.^[^ ^29^, ^30^ ^]^ In this study, we found that osteocyte apoptosis significantly increased following 8 Gy exposure compared with lower doses. This relatively high cellular dose was selected for subsequent studies to establish a damage baseline for evaluating the potential “rescue” effects of vibration.

Radiation exposure induces inflammation and oxidative stress, leading to the generation of ROS. Excessive ROS ultimately results in apoptosis and DNA damage.^[^ ^42^ ^]^ Consistent with previous studies,^[^ ^29^, ^30^ ^]^ we also found that irradiation induced both osteocyte apoptosis and DNA damage. Similar detrimental effects have been observed in mice. A total dose of 16 Gy radiation, administered as two fractions of 8 Gy each, significantly increased the percentage of empty lacunae, an indicator of osteocyte death.^[^ ^8^ ^]^ At the same dose, our collaborator observed an acute reduction in marrow cell viability following irradiation.^[^ ^43^ ^]^ While irradiation reduces bone cell viability, mechanical stimulation has demonstrated its potential to mitigate this effect. Mechanical stimuli, such as loading or stretching, have been shown to prevent osteocyte apoptosis, possibly through activation of the extracellular signal‐regulated kinase (ERK) signaling and nitric oxide production.^[^ ^44^, ^45^ ^]^ Under conditions of cellular damage, mechanical stimulation can also reverse osteocyte apoptosis. For example, mechanical loading mitigated osteocyte apoptosis induced by tumor necrosis factor alpha (TNF‐α),^[^ ^46^ ^]^ and cyclic hydraulic pressure reversed apoptosis caused by serum starvation.^[^ ^47^ ^]^ In this study, LMHF vibration was shown to mitigate osteocyte apoptosis caused by irradiation via the intrinsic apoptotic pathway through *Bcl2l11*. Vibration also downregulated the ROS pathway and the inflammatory response in irradiated osteocytes. These findings expand our understanding of mechanical interventions as radioprotective measures. Intriguingly, a previous study reported that a 12‐h cyclic stretch inhibited DNA damage induced by UV radiation.^[^ ^48^ ^]^ However, vibration did not significantly affect DNA damage induced by gamma radiation. Similarly, a chemical approach using P7C3 also failed to provide radioprotection against DNA damage in irradiated cells.^[^ ^9^ ^]^ Taken together, vibration provides protection against apoptosis but not DNA damage, possibly due to its influence on distinct cellular pathways, as supported by the gene concept network showing that the reversed genes were unrelated to DNA damage. Another possibility is the “outside‐in” theory, in which vibration stimuli initially target signaling pathways or structural components outside the nucleus before affecting the nuclear interior. It is also possible that prolonged vibration treatment might be required to achieve protective effects against DNA damage.

Irradiation not only affects cell viability but also impacts the cytoskeletal integrity, which is critical for maintaining cellular shape, motility, and division. In osteocytes, the cytoskeleton also plays a key role in mechanotransduction.^[^ ^25^ ^]^ Among the three types of cytoskeletal filaments, F‐actin is predominantly located within osteocyte processes, where mechanical stimuli are primarily transmitted.^[^ ^25^ ^]^ Our recent findings demonstrated that vibration tended to increase F‐actin expression in osteocytes,^[^ ^49^ ^]^ further highlighting its significance in osteocyte mechanotransduction. Unfortunately, previous studies have shown that irradiation reduces dendritic length in both MLO‐Y4 osteocytes and primary osteocytes.^[^ ^29^, ^30^ ^]^ Similarly, we observed reduced F‐actin expression, diminished cellular branching, enlarged cell area, and increased nuclear area with irregular morphology in irradiated osteocytes. Notably, vibration only slightly upregulated F‐actin expression in irradiated osteocytes. It is possible that the current vibration settings and timeline were sufficient to observe changes in F‐actin expression, but insufficient to reveal alterations in cell morphology or nuclear features. Alternatively, due to the adaptive capacity of osteocytes to adjust and restore their structure and function, the most informative time points and vibration parameters remain to be determined.

F‐actin in the cytoplasm binds to lamins lining the inner surface of the nuclear envelope through the LINC complex. Although F‐actin and lamins are connected, irradiation affects them differently. We observed decreased F‐actin expression but increased lamin A/C expression in irradiated osteocytes. This observation is consistent with a study showing that irradiation increased lamin A/C expression in both epithelial cells and breast cancer cells 24 h after irradiation.^[^ ^12^ ^]^ Interestingly, this response was reversed in epithelial cells by 72 h.^[^ ^12^ ^]^ Thus, the effect of irradiation on lamin A/C expression is time‐ and cell type–dependent. Lamin A/C, an intermediate filament, maintains nuclear integrity, provides structural support to the nucleus, and assists in DNA repair processes. Loss of A‐type lamins in mouse embryonic fibroblasts induces genomic instability, as evidenced by increased basal DNA damage.^[^ ^50^ ^]^ In irradiated osteocytes, the observed increase in lamin A/C may represent an adaptive response to cope with irradiation‐induced damage within 2 days after exposure, with vibration further enhancing this effect. In bone research, elevated lamin A/C has been shown to promote osteogenic differentiation in mesenchymal stem cells (MSCs).^[^ ^35^ ^]^ Taken together, increased lamin A/C expression may be beneficial; however, as most bone studies have focused on MSCs, the specific role of lamin A/C in osteocytes remains largely unexplored. In MSCs, elevated lamin A/C is also associated with higher nuclear stiffness. Several studies have demonstrated that cells overexpressing lamin A/C have stiffer nuclei that resist deformation and limit migration.^[^ ^51^, ^52^ ^]^ A study on MSCs reported that multiple bouts of vibration (0.7 g, 90 Hz, 20 min × 4 times) were required to increase nuclear stiffness.^[^ ^53^ ^]^ Similarly, in our study, a single bout of vibration was sufficient to upregulate F‐actin expression, whereas at least two bouts were necessary to upregulate lamin A/C in both non‐irradiated and irradiated osteocytes. These findings support the aforementioned “outside‐in” theory, whereby vibration stimuli first target signaling pathways or structural components outside the nucleus, influencing processes such as apoptosis and F‐actin before affecting nuclear function. Further studies are needed to elucidate the underlying mechanisms. Moreover, since mechanical stimuli can also be transmitted through gap junctions, ion channels, focal adhesions, and primary cilia,^[^ ^25^ ^]^ it will be important to investigate whether they are affected by the treatment.

Besides osteocyte viability and cytoskeletal integrity, a critical function of osteocytes is their regulation of other bone cells.^[^ ^26^, ^54^ ^]^ In response to irradiation, osteocytes exhibited increased RANKL expression, thereby promoting bone‐resorptive osteoclast formation,^[^ ^29^, ^30^ ^]^ consistent with our osteocyte‐conditioned media studies. Although vibration did not alter the total number of osteoclasts, it notably reduced the formation of multinucleated giant osteoclasts. This is important because previous studies have demonstrated that, with the same number of cells, larger osteoclasts possess greater bone‐resorbing capacity than smaller ones.^[^ ^55^, ^56^ ^]^ Moreover, when resorption was normalized to the number of nuclei per osteoclast, larger osteoclasts resorbed more bone per nucleus, indicating that as a population, large osteoclasts are more efficient than smaller ones.^[^ ^56^ ^]^ The effects of irradiation have also been demonstrated in vivo, where irradiation increased TRAP+ surface area and osteoclast activity while decreasing the trabecular bone volume in mature mice.^[^ ^43^ ^]^ Importantly, our RNA‐seq data suggested that vibration could alleviate the irradiation‐induced suppression of bone development and growth pathways in osteocytes. Taken together, these findings suggest that vibration may mitigate irradiation‐induced bone loss by modulating osteocyte signaling. Further validation through functional and histological analyses in vivo will be essential.

Beyond their role in bone remodeling, osteocytes also regulate bone metastasis. We and others have shown that conditioned media collected from mechanically loaded osteocytes modulate breast cancer behaviors, with mixed outcomes of either promotion or inhibition.^[^ ^57^, ^58^, ^59^, ^60^ ^]^ These differences may be attributed to variations in shear stress (with 0.8–3 Pa generally considered physiologically relevant) and the type of loading applied (unidirectional versus oscillatory).^[^ ^61^ ^]^ In contrast, conditioned media collected from osteocytes subjected to vibration did not significantly affect cancer migration.^[^ ^23^ ^]^ This lack of effect may be due to differences in the mode of mechanical stimulation and the need for prolonged vibration over multiple days to produce measurable outcomes. Additionally, conditioned‐medium approaches do not fully capture the interactions between bone and cancer cells. To address this limitation, we used a microfluidic platform with a 3D lumen channel to mimic the bone–cancer microenvironment.^[^ ^27^ ^]^ We demonstrated that osteocytes reduced cancer invasion and extravasation under vibration treatment after 3 days.^[^ ^22^ ^]^ Nevertheless, radiotherapy compromised the ability of osteocytes to regulate cancer cells. Irradiated osteocytes promoted invasion and extravasation of non‐irradiated breast cancer cells, whereas vibration reduced cancer extravasation. Under conditions where all cells in the microfluidic device were irradiated, irradiation disrupted the HUVEC barrier but still reduced cancer extravasation overall. Vibration further decreased breast cancer invasion and extravasation when co‐cultured with osteocytes, suggesting its potential as an adjunct to radiotherapy. Importantly, this vibration effect was not observed in the absence of osteocytes, emphasizing their critical role in regulating breast cancer metastasis. Recently, Wang et al. also approached the problem from the opposite perspective, showing that conditioned media collected from breast cancer cells influenced osteocyte function.^[^ ^62^ ^]^ Again, conditioned‐medium experiments are limited in that they only capture one‐way communication. By contrast, our microfluidic platform better mimics the bidirectional crosstalk that occurs in vivo. Both the current study and our previous work have demonstrated that osteocytes regulate breast cancer extravasation under mechanical stimulation,^[^ ^22^, ^27^ ^]^ suggesting that osteocyte function is maintained in the presence of breast cancer cells.

The mechanisms underlying osteocyte regulation of breast cancer behavior have recently been investigated, but mixed results have been reported. Some studies showed that osteocytes promoted breast cancer progression via TNF‐α^[^ ^63^ ^]^ or by secreting chemokines such as CXCL1/2 under mechanical loading.^[^ ^58^ ^]^ In contrast, other studies demonstrated a tumor‐suppressive role, showing that mechanically loaded osteocytes inhibited breast cancer migration via Cx43 hemichannels.^[^ ^59^ ^]^ Follow‐up research reported that antibody activation of Cx43 in osteocytes induced ATP release, which activated the P2X7 receptor and thereby inhibited the growth and migration of breast cancer cells.^[^ ^64^ ^]^ Consistent with this inhibitory function, chemical activation of Wnt signaling pathway in osteocytes using BML284 enhanced their tumor‐suppressive activity by reducing tumor progression in mice and downregulating tumor‐promoting genes.^[^ ^65^ ^]^ Building on these mechanistic findings, we examined various tumor‐regulating pathways in osteocytes. Notably, irradiated osteocytes subjected to vibration treatment suppressed cancer behaviors, possibly through mechanical modulation of the Wnt signaling pathway. While Wnt signaling is tumorigenic in cancer cells,^[^ ^66^ ^]^ it supports tumor‐suppressive signaling in osteocytes.^[^ ^65^ ^]^ Importantly, inhibition of Wnt signaling in irradiated osteocytes abolished the vibration‐induced downregulation of tumor‐promoting genes such as *Ccnd1* and *Hmga2*. *Ccnd1* is implicated in breast cancer growth and invasion, whereas *Hmga2* promotes epithelial‐to‐mesenchymal transition (EMT) and contributes to breast cancer metastasis.^[^ ^67^, ^68^ ^]^ These findings highlight the tumor‐suppressive role of Wnt signaling in osteocytes under mechanical stimulation.

Despite these promising findings, our study has several limitations. First, although the MLO‐Y4 osteocyte‐like cell line is widely accepted as a model for osteocyte mechanobiology, it has known limitations. For example, these cells lack detectable expression of key markers such as sclerostin (Sost), which is important for signaling to osteoblasts; however, osteoblasts were not the focus of this study. Future work will include osteoblasts together with primary osteocytes to better understand bone remodeling under irradiation and vibration. In addition, our use of both human (HUVECs and MDA‐MB‐231) and murine (MLO‐Y4) cell lines may raise concerns about cross‐species variation. However, this approach aligns with common practice, as MDA‐MB‐231 cells are frequently used in murine models of breast cancer. HUVECs are also widely employed to establish endothelial barriers in MDA‐MB‐231 breast cancer metastasis studies, making our findings comparable to those of others.^[^ ^69^, ^70^ ^]^ Second, triple‐negative breast cancer (TNBC) is a highly invasive subtype, with 46% of TNBC patients developing distant metastasis^[^ ^71^ ^]^; hence, it is suitable for our extravasation studies. Moreover, its lack of therapeutic targets often necessitates radiotherapy, making it relevant to the context of this study. However, TNBC is less prevalent than estrogen receptor (ER)‐positive breast cancer, which comprises about 80% of cases, but ER+ breast cancer is considered less aggressive.^[^ ^72^, ^73^ ^]^ Our previous study reported that MCF‐7 ER+ breast cancer cells did not migrate significantly toward osteocyte‐conditioned media.^[^ ^57^ ^]^ Future studies should incorporate other cancer cell lines to broaden the applicability of our findings. Lastly, our study primarily focuses on in vitro models. Although we used the microfluidic platform to mimic the bone–cancer microenvironment, it cannot fully capture the complexity of the bone physiology and metastatic progression in patients. Further steps will include validating these findings in vivo and performing mechanistic analyses, such as histological staining of relevant mediators to substantiate pathway involvement (i.e., intrinsic signaling and Wnt signaling). Additionally, the optimal parameters of LMHF vibration, including frequency, magnitude, and duration, remain to be determined to maximize the effects and facilitate translation into preclinical models and, ultimately, clinical trials.

## 4 Conclusion

While chemical‐based radioprotection strategies, such as P7C3^[^ ^9^ ^]^ and PTH1‐34,^[^ ^8^ ^]^ have garnered considerable attention, our study offers a novel perspective by exploring LMHF vibration as a mechanical approach to mitigating radiotherapy‐induced complications. We found that LMHF vibration effectively reduced osteocyte apoptosis, upregulated cytoskeletal markers, and restored osteocyte regulation of breast cancer metastasis following irradiation. Our findings provide mechanistic insight into the cellular basis of irradiation‐induced bone damage and highlight the potential of LMHF vibration as a therapeutic strategy for preserving bone health and optimizing cancer treatment outcomes.

## 5 Experimental Section

### Cell Culture—MLO‐Y4 Cells

A murine osteocyte‐like cell line (a gift from Dr. Lynda Bonewald, Indiana University) was cultured on Petri dishes or glass slides coated with 0.15 mg mL^−1^ type I rat‐tail collagen (354236, Corning), diluted in 0.02 N acetic acid (A6283, Sigma‐Aldrich). Cells were grown to 80% confluence in 94% v/v α‐MEM basal medium (12571063, Gibco), supplemented with 2.5% fetal bovine serum (FBS, 12483‐020, Gibco), 2.5% calf serum (CS, 16010‐159, Gibco), and 1% penicillin–streptomycin (P/S, 15140122, Gibco).

### Cell Culture—RAW 264.7 Cells

The RAW 264.7 (TIB‐71, ATCC) cell line was maintained in 87% DMEM (D5671, Sigma‐Aldrich), supplemented with 10% FBS, 1% P/S, and 2% L‐glutamine (25030081, Gibco). Cells were harvested using cell scrapers for subculturing and seeding.

### Cell Culture—HUVECs

Human umbilical vein endothelial cells (HUVECs) (a gift from Dr. Craig Simmons, University of Toronto) were cultured in EndoMax basal medium (301‐010‐CL, Wisent) supplemented with 2% EndoMax growth supplement (301‐013‐XL, Wisent), 10% FBS, and 1% P/S.

### Cell Culture—MDA‐MB‐231 Cells

A human metastatic TNBC cell line (HTB‐26, ATCC) was grown in F‐12K basal medium (21127022, Gibco) supplemented with 10% FBS and 1% P/S.

### Irradiation

The irradiator used was a Gammacell 1000 Elite with a Cs‐137 gamma source. During cell passaging, cells collected in tubes were irradiated immediately before seeding. The dose rate at chamber wall was 0.077 Gy s^−1^ (e.g., 104 s for an 8 Gy dose), and it was calibrated annually by Environmental Health and Safety at the University of Toronto.

### Low‐Magnitude High‐Frequency Vibration

The custom‐made vibration platform generated vertical, sinusoidal motion with a magnitude of 0.3 g and a frequency of 60 Hz (Figure S1, Supporting Information). The platform was operated inside an incubator at 37 °C and 5% CO_2_, providing stable vibration while enabling real‐time monitoring of the magnitude, as described in previous studies.^[^ ^22^, ^49^, ^74^ ^]^

### Apoptosis Assay

At 2 days post‐irradiation (8 Gy), irradiated or non‐irradiated osteocytes underwent LMHF vibration (0.3 g, 60 Hz, 1 h), during which cells were in fresh culture medium. Following vibration, cells were incubated for 1 h at 37 °C and 5% CO_2_. Apoptosis was assessed by incubating the cells with 5% APOPercentage dye (A1000, Biocolor) in culture medium for 30 min, followed by a PBS wash. Random images were captured under a light microscope at 20× magnification. Apoptotic cells, which stained pink, were counted, and the number of apoptotic cells was normalized to the total cell count per field.

### Immunofluorescence Staining—DNA Damage

At 2 days post‐irradiation (8 Gy), irradiated or non‐irradiated osteocytes underwent LMHF vibration (0.3 g, 60 Hz, 1 h) or remained static in fresh culture medium. Following vibration, cells were incubated for 1 h at 37 °C and 5% CO_2_. Cells were fixed with 4% formaldehyde for 15 min and blocked with 5% normal goat serum (5425, Cell Signaling) and 0.3% Triton X‐100 in PBS for 1 h. They were then incubated overnight at 4 °C with a phospho‐histone H2A.X (Ser139) antibody (2577, Cell Signaling) diluted 1:800 in 1% bovine serum albumin (BSA) with 0.3% Triton X‐100 in PBS. The next day, an anti‐rabbit Alexa Fluor 488–conjugated secondary antibody (4412, Cell Signaling) was added at 1:600 in 1% BSA with 0.3% Triton X‐100 in PBS for 1.5 h. Cells were counterstained with DAPI (MBD0015, Sigma‐Aldrich) at 1:1000 in PBS for 10 min. Random images were captured using a confocal microscope (Nikon A1) at 40× magnification. Quantification of γH2AX foci was performed using ImageJ (v2.14.0).

### Immunofluorescence Staining—Lamin A/C and F‐Actin

At 2 days post‐irradiation (8 Gy), irradiated or non‐irradiated osteocytes underwent LMHF vibration (0.3 g, 60 Hz, 1 h day^−1^) or remained static for 1 or 2 days, during which cells were in fresh culture medium. The cells were then fixed with 4% formaldehyde for 15 min. Cells were blocked with 5% normal goat serum and 0.3% Triton X‐100 in PBS for 1 h. They were incubated overnight at 4 °C with an anti‐mouse Alexa Fluor 647–conjugated lamin A/C antibody (4C11, Cell Signaling) diluted 1:200 in 1% BSA and 0.3% Triton X‐100 in PBS. For F‐actin staining on the next day, cells were permeabilized with 0.1% Triton X‐100 for 5 min and incubated with phalloidin–iFluor 488 reagent (ab176753, Abcam) at 1:1000 dilution in 1% BSA for 1 h. Cells were then counterstained with DAPI at 1:1000 in PBS for 10 min. Random images were captured using a confocal microscope (Leica Stellaris 5) at 25× magnification. Fluorescence intensity, cellular branching, cell area, nuclear area, and nuclear form factor were analyzed using CellProfiler (v4.2.6).

### Immunofluorescence Staining—VE‐Cadherin

Irradiated or non‐irradiated HUVECs in a microfluidic platform underwent LMHF vibration (0.3 g, 60 Hz, 1 h day^−1^) or remained static for 3 days. The cells were fixed with 4% formaldehyde for 15 min and permeabilized with 0.3% Triton X‐100 for 5 min. Cells were then blocked with 5% normal donkey serum and 1% BSA for 1 h. They were incubated overnight at 4 °C with 3 µg mL^−1^ of VE‐cadherin antibody (AF938‐SP, Cedarlane) in 1% BSA. The next day, an anti‐goat Alexa Fluor 488–conjugated secondary antibody (A32814, ThermoFisher) was added at 1:100 in 1% BSA for 1.5 h. Cells were then counterstained with DAPI at 1:1000 in PBS for 10 min. Random images were captured using a confocal microscope (Leica Stellaris 5). HUVEC perimeter and VE‐cadherin mean fluorescence intensity were analyzed using CellProfiler (v4.2.6).

### Osteoclastogenesis

Irradiated or non‐irradiated osteocytes were subjected to LMHF vibration (0.3 g, 60 Hz, 1 h) in fresh culture medium, followed by incubation for 3 h before collecting the osteocyte‐conditioned media. RAW 264.7 osteoclast precursors were seeded in a 24‐well plate in RAW medium (Day 0). After 48 h (Day 2), cells were cultured with the osteocyte‐conditioned media (mixed with RAW medium at a 1:1 ratio) daily until Day 4. An additional 20 ng mL^−1^ RANKL (462‐TR‐010, R&D Systems) was added to induce osteoclastogenesis along with the treatment. On Day 5, the medium was replaced with pure RAW medium. On Day 6, the osteoclasts were fixed and stained with TRAP. Random images were captured under a light microscope for osteoclast quantification, and the osteoclasts were identified as TRAP+ multinucleated cells.

### Microfluidic Co‐Culture Platform—Osteocyte and Lumen Microenvironment

A microfluidic co‐culture platform with a previously established multi‐layer design^[^ ^22^, ^27^ ^]^ was employed to investigate breast cancer invasion and extravasation. The platform design was first patterned onto a photomask and then transferred onto a silicon wafer spin‐coated with SU‐8 2050 (Y111072, MicroChem) and 2075 (Y111074, MicroChem) via UV exposure. The resulting silicon wafer, containing imprinted designs of the osteocyte, side, and lumen channels, served as the master mold for polydimethylsiloxane (PDMS) casting. PDMS (DOW) was mixed with its curing agent (DC4019862, DOW) at a 10:1 ratio and cured at 60 °C overnight. The cured PDMS structure was then cut and bonded to a 75 mm × 50 mm glass slide using oxygen plasma treatment. The device was sterilized by rinsing with 70% ethanol, followed by PBS washes. Gel coating followed previously published procedures.^[^ ^22^, ^27^ ^]^ Briefly, osteocyte channels were coated with a layer of 0.15 mg mL^−1^ type I rat‐tail collagen for 1 h. All channels were then coated with a 100 µg mL^−1^ fibronectin solution (F1141‐2MG, Sigma‐Aldrich) for 40 min at 4 °C. A hydrogel solution, with final concentrations of 5.5 mg mL^−1^ of type I rat‐tail collagen (354249, Corning) and 2.5 mg mL^−1^ of Matrigel (354230, Corning), was then slowly loaded into the lumen channel and removed within 30 s. The coated device was incubated at 37 °C for 1 h to solidify the 3D hydrogel lumen. After gelation, all channels were filled with media and prepared for cell seeding.

### Microfluidic Co‐Culture Platform—Irradiated Osteocyte Regulation of Breast Cancer Metastasis

HUVECs labeled with CellTracker^TM^ Orange (Ex: 541 nm, Em: 565 nm; Invitrogen) were seeded at 2000K cells mL^−1^ per side of the lumen channel in different orientations to form a circular layer lining the lumen channel.^[^ ^75^ ^]^ Irradiated or non‐irradiated MLO‐Y4 osteocytes were seeded at 1500K cells mL^−1^ into the osteocyte channel. After 5 h, once HUVECs and osteocytes had attached, fluorescent‐labeled MDA‐MB‐231 cancer cells (CellTracker^TM^ Green) were seeded at 4000K cells mL^−1^ into the lumen channel. Cells in the platform received vibration (0.3 g, 60 Hz, 1 h day^−1^ for 3 days) or remained static. Growth media in both channels were replenished every 24 h. Cancer invasion (without HUVECs) or extravasation (with HUVECs) was fluorescently imaged after three vibration treatments on Day 4. Distance measurements were performed in ImageJ (v2.14.0).

### Microfluidic Co‐Culture Platform—Breast Cancer Metastasis Following Irradiation

Irradiated or non‐irradiated cells, including HUVECs, MLO‐Y4 osteocytes, and MDA‐MB‐231 cancer cells were seeded as described above. Cells in the platform received vibration (0.3 g, 60 Hz, 1 h day^−1^ for 3 days) or remained static. Growth media in both channels were replenished every 24 h. Cancer invasion (without HUVECs) or extravasation (with HUVECs) was fluorescently imaged after three vibration treatments on Day 4. Distance measurements and percentages of metastasis were assessed in ImageJ (v2.14.0).

### RNA‐Seq—Sample Preparation

At 2 days post‐irradiation (8 Gy), irradiated or non‐irradiated osteocytes underwent LMHF vibration (0.3 g, 60 Hz, 1 h) in fresh culture medium. Total RNA was extracted immediately after vibration treatment using an RNA extraction kit (74104, Qiagen), according to the manufacturer's instructions. Library construction and RNA‐seq were performed by Azenta Life Sciences. Sequencing was conducted using an Illumina sequencer in 150 bp paired‐end mode, and the quality of clean reads was assessed using FastQC. Further analysis was conducted using R (v4.5.1).

### RNA‐Seq—Principal Component Analysis

Data quality was assessed using principal component analysis (PCA) to evaluate sample similarity. PCA was performed in R using the plotPCA function from the DESeq2 (v1.48.1) package, which extracts the top principal components based on the variance‐stabilizing transformation of the count data.

### RNA‐Seq—Differential Gene Expression with Multiple‐Group Analysis

Differential gene expression analysis was performed using DESeq2 in R, employing the Wald test for significance testing. Multiple‐group comparisons were conducted among non‐irradiated osteocytes (N), irradiated osteocytes (R), and irradiated osteocytes treated with vibration (RV), with irradiated osteocytes (R) as the reference. Differentially expressed genes (DEGs) were considered significant if the adjusted p‐value was < 0.05 and the absolute log2 fold change was > 0.1. Gene symbols were mapped using the org.Mm.eg.db (v3.21.0) database in R.

### RNA‐Seq—Gene Ontology Analysis

Significant DEGs were classified into gene ontology terms using the clusterProfiler package (v4.16.0) in R.

### RNA‐Seq—Gene Set Enrichment Analysis

Changes in gene expression within predefined gene sets were analyzed to compare irradiated osteocytes (R) and irradiated osteocytes after vibration treatment (RV) using GSEA software (v4.3.3). The signal‐to‐noise ratio was used as the ranking metric.

### RNA‐Seq—STRING Analysis

Protein–protein interactions (PPIs) were analyzed using the Search Tool for the Retrieval of Interacting Genes/Proteins (STRING, v12.0). The interaction score was computed based on active interaction sources, including text mining, experiments, databases, co‐expression, neighborhood, gene fusion, and co‐occurrence.

### Quantitative Polymerase Chain Reaction

At 2 days post‐irradiation (8 Gy), irradiated or non‐irradiated osteocytes underwent LMHF vibration (0.3 g, 60 Hz, 1 h) in fresh culture medium. Total RNA was extracted immediately after vibration treatment using the RNA extraction kit, according to the manufacturer's instructions. After DNA digestion with DNase (EN0521, ThermoFisher), RNA was reverse transcribed using SuperScript III reverse transcriptase (18080‐044, Invitrogen). qPCR was performed using LightCycler 480 SYBR Green I Master Mix (04707516001, Roche) and gene‐specific primers (Table S1, Supporting Information), with 18S serving as the housekeeping gene. Relative mRNA levels were calculated using the ΔCt method.^[^ ^76^ ^]^

### Wnt Pathway Inhibition

At 2 days post‐irradiation (8 Gy), irradiated osteocytes were treated with the Wnt pathway inhibitor XI, CCT036477 (681674, Sigma‐Aldrich) at 20 µM for 4 h.^[^ ^37^ ^]^ The cells were then incubated in fresh media for 1 h before undergoing vibration treatment (0.3 g, 60 Hz, 1 h). Total RNA was extracted for qPCR as described above.

### Statistical Analysis

All experiments were independently replicated at least three times (N = 3), with a minimum of four technical replicates (n = 4) per condition in each experiment, except for RNA‐seq data. The specific sample size for each statistical analysis is provided in the figure captions. For the microfluidic experiments, there were six microfluidic devices per platform, with each device containing five side channels. Thus, a technical replicate of n = 30 side channels was prepared per condition; however, the actual number varied depending on the quality of plasma bonding and hydrogel formation. Data are expressed as the mean ± standard deviation (SD). Statistical significance was evaluated using Student's t‐test or one‐way analysis of variance (ANOVA) with Tukey's correction for multiple comparisons, as appropriate (Prism 9, GraphPad). Differences were considered significant if the *p*‐value was < 0.05 (*p < 0.05, **p < 0.01, ***p < 0.001, ****p < 0.0001).

## Conflict of Interest

The authors declare no conflict of interest.

## Supporting information



Supporting Information

## Data Availability

The data that support the findings of this study are available from the corresponding author upon reasonable request.
