# Supplementary material for: Vibration‐Mediated Recovery of Irradiated Osteocytes and Their Regulatory Role in Breast Cancer Bone Metastasis
Source: Adv Healthc Mater. 2025 Oct 15;15(4):e01689. doi: 10.1002/adhm.202501689 (PMC12836450; doi:10.1002/adhm.202501689)
Supplement: Supplementary file 1 — Supporting Information [file ADHM-15-0-s001.docx]

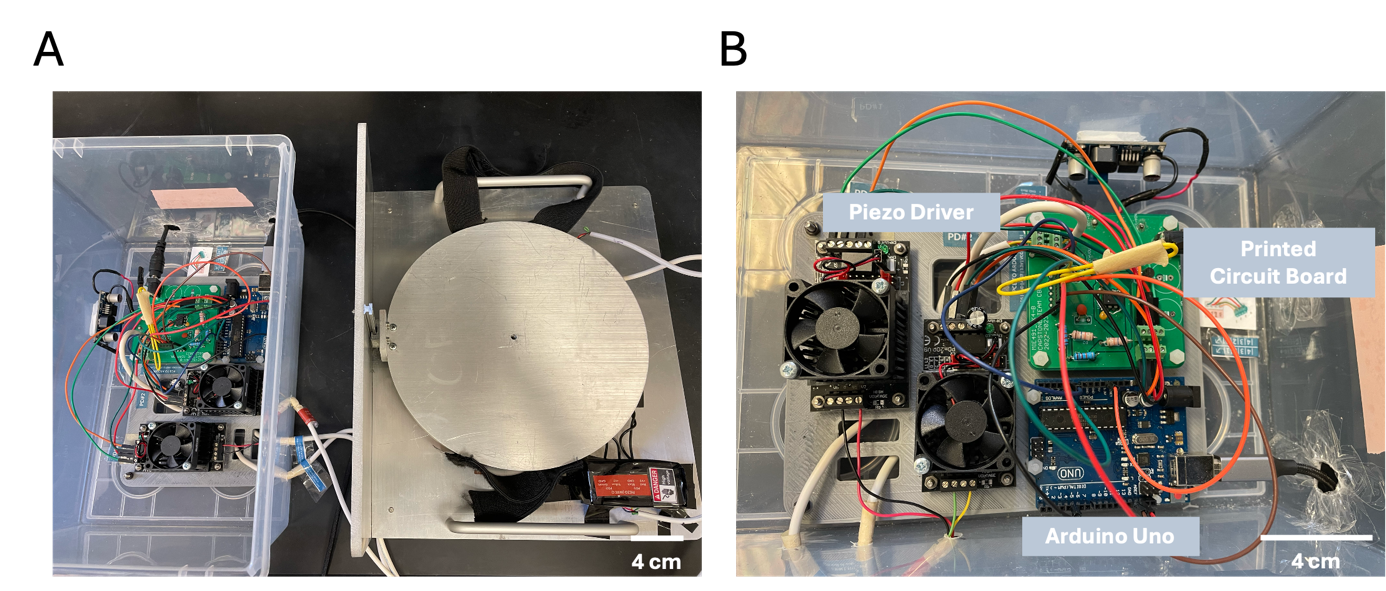
**Supporting Information**

**Figure S1** Vibration platform. **A)** Top view of the vibration platform. **B)** The microcontroller (Arduino Uno) is connected to a printed circuit board and two piezo drivers. Scale bar = 4 cm.

**
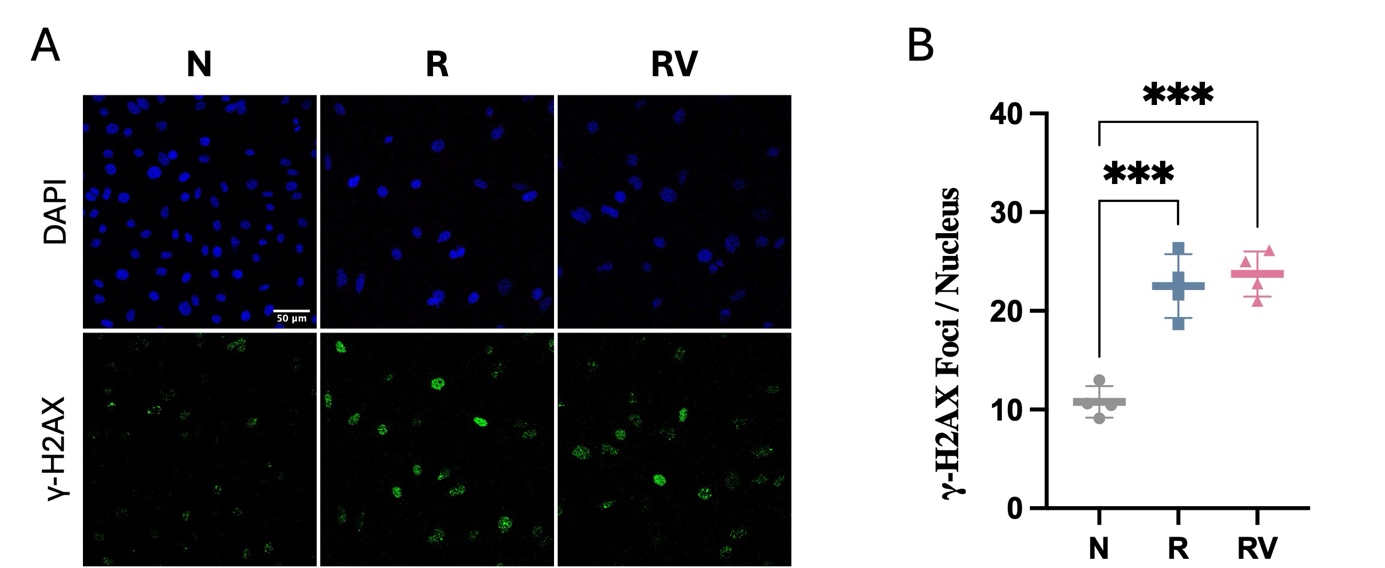
Figure S2** Osteocytes DNA damage. **A)** Immunofluorescence staining of γH2AX and DAPI in osteocytes. All images share the same scale (scale bar = 50 μm). **B)** Number of γH2AX foci per nucleus in osteocytes. Data presented as mean ± SD, n = 4. Significance was calculated using one-way ANOVA with Tukey’s correction (P < 0.05). N: non-irradiated osteocytes, R: irradiated osteocytes, RV: irradiated osteocytes treated with vibration.

**
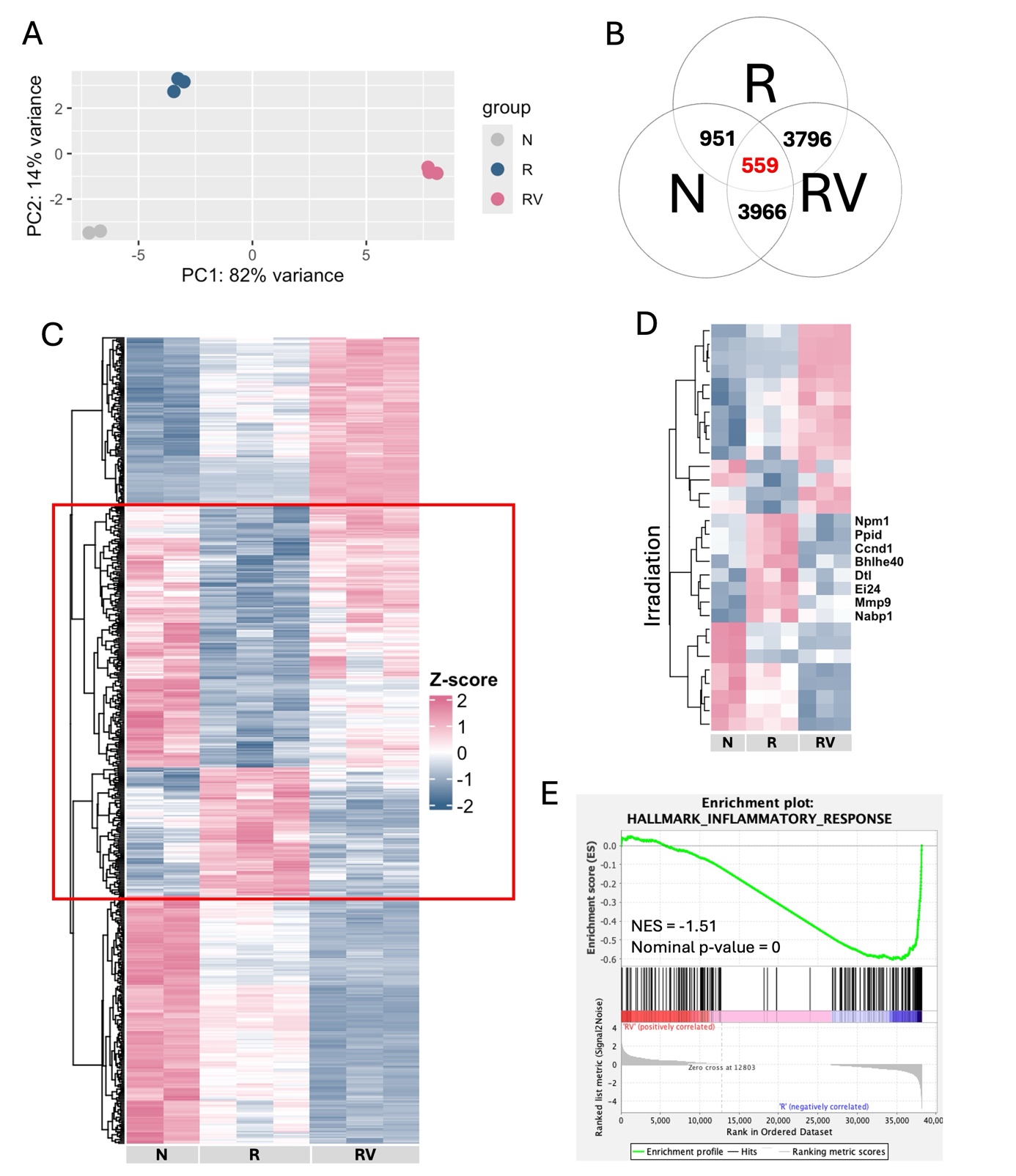
Figure S3** RNA-seq data analysis in osteocytes. **A)** Principal component analysis (PCA) plot showing sample distribution based on the top two principal components (PC1 and PC2), which capture the largest variance in the dataset. **B)** Venn diagram illustrating the overlap among differentially expressed genes (DEGs). **C)** Heatmap displaying the expression profiles of DEGs across multiple treatment groups, with R as the reference. Genes outlined in the red box represent irradiation-induced changes that were reversed by vibration. **D)** Heatmap of DEGs associated with the irradiation response. Labeled genes: irradiation-induced expression changes that were reversed by vibration. **E)** Gene set enrichment analysis (GSEA) of the inflammatory response in RV relative to R. NES: normalized enrichment score. N: non-irradiated osteocytes, R: irradiated osteocytes, RV: irradiated osteocytes treated with vibration.

**
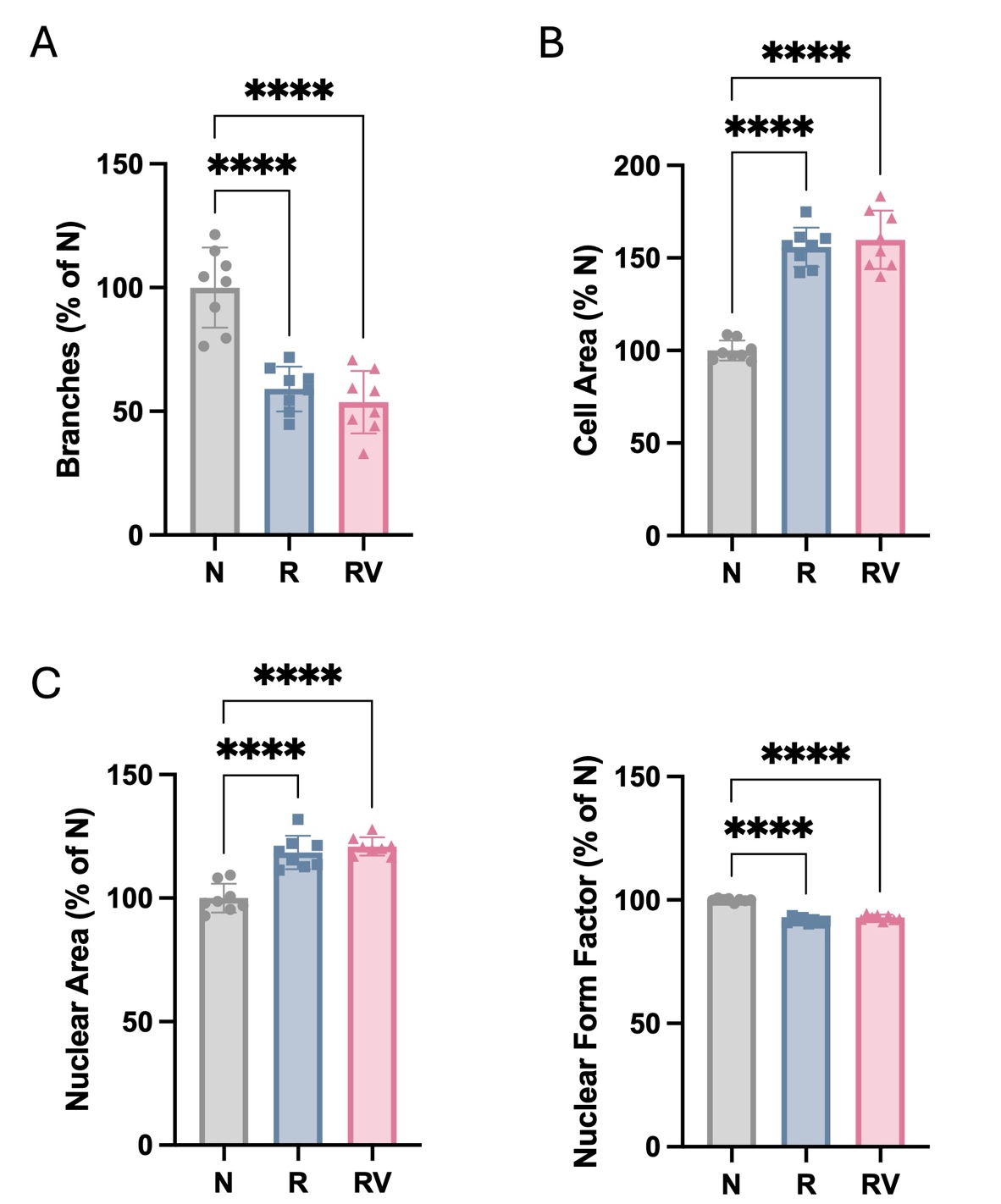
Figure S4** Cellular morphology and nuclear features analyzed based on F-actin and DAPI staining. **A)** Quantification of cell branches, **B)** cell area, **C)** nuclear area, and nuclear form factor, all normalized to the non-irradiated control. Data presented as mean ± SD, n = 8. Significance was calculated using one-way ANOVA with Tukey’s correction (P < 0.05). N: non-irradiated osteocytes, R: irradiated osteocytes, RV: irradiated osteocytes treated with vibration.

**Figure S
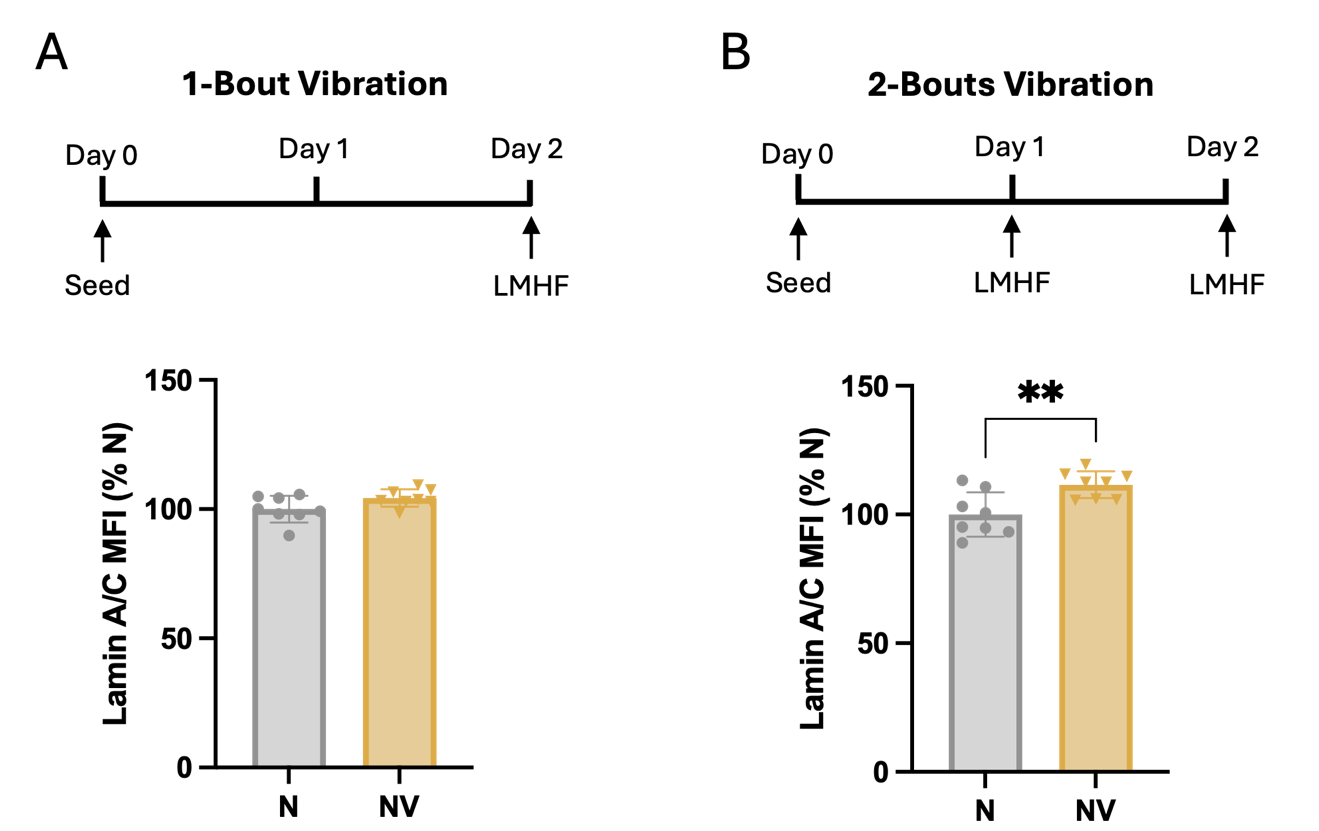
5** Nuclear envelope in non-irradiated osteocytes subjected to vibration. **A)** Timeline of one-bout vibration and mean fluorescent intensity of lamin A/C–stained nuclear envelope. **B)** Timeline of two-bout vibration and mean fluorescent intensity of lamin A/C–stained nuclear envelope. Data presented as mean ± SD, n = 4. Significance was calculated using Student’s t-test (P < 0.05). N: osteocytes in static, NV: osteocytes treated with vibration.

**
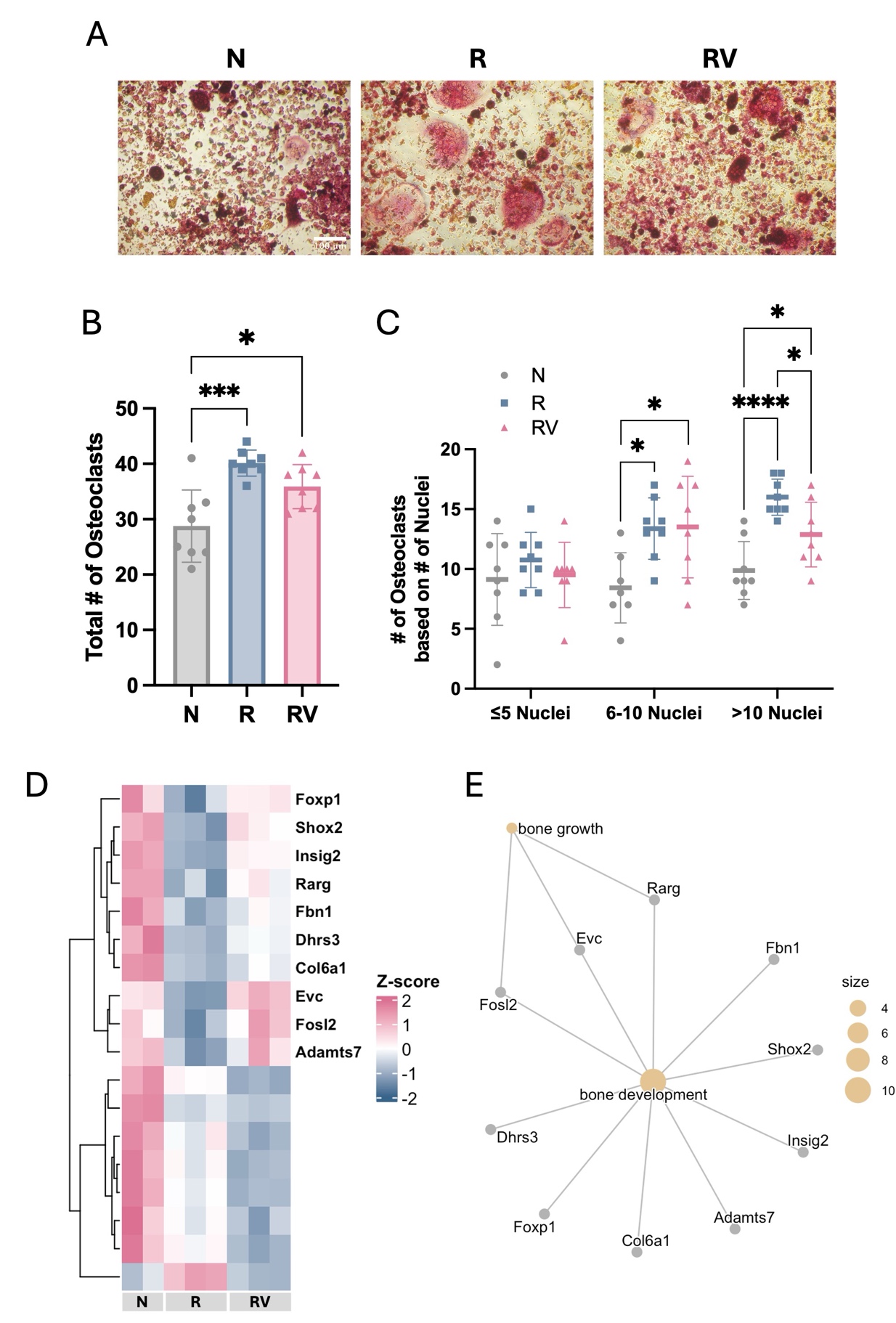
Figure S6** Irradiated osteocyte regulation of bone development. **A)** Representative image of TRAP+ RAW 264.7 osteoclasts cultured in osteocyte conditioned media. All images share the same scale (scale bar = 100 μm). **B)** Total number of osteoclasts after 6 days of differentiation and **C)** osteoclast classification based on the number of nuclei. Data presented as mean ± SD, n = 8. Significance was calculated using one-way ANOVA with Tukey’s correction (P < 0.05). **D)** Heatmap of differentially expressed genes (DEGs) related to bone development and bone growth. Labeled genes: irradiation-induced expression changes that were reversed by vibration. **E)** Gene concept network related to the labeled genes. N: non-irradiated osteocytes, R: irradiated osteocytes, RV: irradiated osteocytes treated with vibration.

**Table S1** Gene-specific primers

|  | **Forward (5'-3')** | **Reverse (5'-3')** | **Product Size (bp)** |
| --- | --- | --- | --- |
| **18S** | GAGAAACGGCTACCACATCC | CCTCCAATGGATCCTCGTTA | 158 |
| **Ccnd1** | TGGATGCTGGAGGTCTGTGA | AACTTCTCGGCAGTCAAGGG | 185 |
| **Hmga2** | GCAGCCCAGAAGAAAGCAGA | GTCTCCTGAGCAGGCTTCTT | 101 |
